# Advance on establishment of pathological model of ulcerative colitis

**DOI:** 10.3389/fvets.2025.1618260

**Published:** 2025-08-26

**Authors:** Lan Yang, Han Gao, Dacheng Liu

**Affiliations:** College of Veterinary Medicine, Inner Mongolia Agricultural University, Hohhot, China

**Keywords:** Crohn’s disease, ulcerative colitis, animal models, etiology, pathogenesis

## Abstract

Inflammatory bowel disease (IBD), comprising Crohn’s disease (CD) and ulcerative colitis (UC), is a chronic, relapsing inflammatory disorder of the gastrointestinal tract with multifactorial etiology. The etiology and pathogenesis of ulcerative colitis are diverse, so it is crucial to explore the pathogenesis through animal models. Selecting appropriate animal models is critical for advancing our understanding of UC pathogenesis and therapeutic strategies. This review discusses various UC animal models and compares their characteristics in relation to disease mechanisms and therapeutic research.

## Introduction

Ulcerative colitis (UC) is a chronic inflammatory disease of the colon that typically begins in the rectum and progresses proximally in a continuous pattern, affecting the mucosal layer of the colon (1). Patients usually present with abdominal pain, diarrhea, and bloody stools, with the latter being a hallmark of UC. The development of appropriate animal models is essential to understand the pathogenesis of UC and evaluate new treatments.

### Exploration of the pathogenesis of disease

Experimental models of ulcerative colitis can simulate the development and progression process of the disease, enable detailed investigation of the role and interrelationship of immune response, intestinal microbiota, genetic susceptibility and environmental factors in the pathogenesis of UC. For example, dextran sulfate sodium DSS—induced UC models allow researchers to study pathological changes in intestinal tissue and cytokine responses to better understand mucosal barrier disruption and inflammatory activation (1, 2). The neurotransmitters secreted by intestinal neurons, such as 5-hydroxytryptamine, also modulate mucosal barrier function and contribute to gut inflammation (3).

### Facilitating drug development

The animal models of ulcerative colitis can be constructed to provide an effective platform for drug development, which can be used to test the efficacy and safety of various new drugs, accelerating the drug development process, and improving the success rate of development. For example, a model of ulcerative colitis established by a team from Kyoto University in Japan using human induced pluripotent stem cell-derived colonoid organs provides a promising platform for testing therapeutics such as tofacitinib (4).

### Toward precision medicine

Different animals or patients with ulcerative colitis may have individual differences. Animal models can simulate distinct UC subtypes and disease stages, offering valuable insights for designing personalized treatment strategies. This approach supports the development of precision medicine aimed at improving treatment efficacy and patient prognosis.

## Pathogenesis of ulcerative colitis

### Environmental factors

In some developed countries, the incidence of UC has shown a significant increasing trend, which may indicate that environmental triggers are an important influencing factor in the development of UC (5). Lifestyle changes, dietary inconsistencies, and use of certain medications such as nonsteroidal antiinflammatory drugs (NSAIDs) and antibiotics are considered contributing factors to UC (6). Interestingly, smoking has been observed to reduce the incidence of UC, possibly due to the effects of carbon monoxide and nicotine on mucosal immune responses (7). In addition, there is evidence that the pathogenic factors of IBD show a clear negative correlation with breastfeeding methods, reproduction modes, and individual animal factors (8).

### Gut microbiota

The gut microbiome is a relatively complex community that lives in symbiosis with its host in the gastrointestinal tract. The development of multiple diseases may be associated with dysbiosis of the gut microbiome. For example, dysbiosis of the gut microbiota can damage the intestinal mucosal barrier, disrupting intestinal permeability, enabling pathogenic microorganisms to exert inflammatory effects through the barrier, and thus leading to the development of UC. Moreover, an imbalance in the gut microbiota can cause some harmful bacteria to proliferate massively. This has been linked to chronic inflammation and the release of pro-inflammatory factors, increasing the risk of developing UC. For patients with UC, the most striking change is an imbalance in the gut microbiota, characterized by a decrease in the abundance of Firmicutes and Bacteroidetes (9). In patients with UC, the damage of the intestinal mucosal layer causes bacterial invasion of the submucosa and massive proliferation of bacteria in the submucosa, leading to inflammation (10, 11). The inflammation results in further damage to the mucosal layer of the intestinal wall, more bacteria invade and cause the submucosal layer to rupture, producing a vicious cycle (10). The Human Microbiome Project found that the levels of short chain fatty acids (SCFAs), *Ruminococcaceae* and *Lachnospiraceae* in the gut of UC patients was markedly reduced, and the abundance of pro-inflammatory microorganisms, such as Enterobacteriaceae, was significantly increased (12, 13). Therefore, the imbalance of gut microbiota is a major influencing factor in the development of UC.

### Genetic factors

While genetic susceptibility contributes to UC development, no single mutation fully accounts for disease onset, as the recombinant protein IL23 receptor (IL23R), the intracellular receptor NOD2 and the leukocyte antigen HLA have the strongest genetic effects (14). Relevant studies have shown that the development of UC is associated with genetic susceptibility, and the incidence of relatives and twins of UC patients is significantly higher than that of other normal population. These studies have helped us to better understand the pathogenesis of UC. Mayberry and other scholars found in 1980 that the children and siblings of UC patients had a 30-fold higher risk of developing UC later compared with normal people (15). Monsén et al. (16) also found a significantly higher incidence of UC in immediate family members of UC patients compared with the others in their families. To explore the genetic factors of UC, a genome-wide association study (GWAS) or meta-analysis was performed to formulate relevant immunoarrays to pinpoint the relevant susceptibility loci of UC, which can help us identify relevant genetic variations in IBD and UC (14, 17, 18).

### Abnormal immune response

A hallmark of UC histology is the presence of a ‘crypt abscess’, which is formed by neutrophil infiltration into the colonic crypt (6). During the inflammatory process, neutrophils first pass through the colonic epithelium and eventually undergo apoptosis in the crypts of the colon (19). Neutrophils may survive the inflammation by the hypoxia-inducible factor HIF-1α, but prolonged neutrophil survival may lead to excessive release of reactive oxygen species and pro-inflammatory mediators (20–22). Calprotectin is present in neutrophils. It is released through the patient’s blood or feces and serologically reacts with the patient’s perinuclear anti-neutrophil antibodies (23–25). Thus, after the onset of the disease, inflammatory neutrophils and monocytes and their pro-inflammatory factors generate an inflammatory response that promotes the pathological process (6, 26). Such an environment contributes to the subsequent neutrophils, monocytes, maintenance of their functional and survival efficiency, and affects the patient’s ability to resolve the inflammation and to redress their homeostasis (27, 28).

### Mitochondrial abnormalities

The latest research on ulcerative colitis is a direct exploration of the intestinal mucosa (29, 30). Some experts found that the expression of genes responsible for energy production in mitochondria (gene encoding mitochondrial oxidative phosphorylation components) was significantly reduced, which suggests that abnormalities in mitochondria can lead to the development of ulcerative colitis (6, 27). Mitochondria are special membrane-bound intracellular compartments that are considered the “powerhouse of the cell,” as they produce a large amount of ATP that is used to power the cell’s biochemical reactions. Previous studies have demonstrated that the mitochondria are involved in the inflammatory response (31, 32), and that loss of mitochondrial function or mitochondrial dysfunction is also an important factor in the development of ulcerative colitis. The studies on mitochondrial function have found that mitochondria are usually located in a specific environment (33, 34), and mitochondrial dysfunction may trigger oxidative stress, reduce ATP production, and release mitochondrial DNA, which acts as a danger-associated molecular pattern (DAMP), amplifying inflammation (35–37). The above studies help us understand the relationship between mitochondria and ulcerative colitis and find a new approach to treat ulcerative colitis, for example, anti-oxidative stress therapy targeting the mitochondria of UC patients.

## Extraintestinal manifestations of UC

### Types of extraintestinal manifestations

Extraintestinal manifestations (EIMs) are the main factors that lead to complications and decrease the chance of survival in patients (38). Kilic et al. (39) categorized EIMs into two groups: those directly associated with intestinal inflammation (e.g., arthritis, iritis) and those considered independent autoimmune manifestations (e.g., primary sclerosing cholangitis).

### Characteristics of extraintestinal manifestations

Due to cross-reactivity of antigens, the gut microbiota may trigger systemic immune responses that affect extraintestinal sites such as skin and joints (40). Disruption of the intestinal barrier allows bacterial antigens to trigger systemic immune responses, including cross-reactivity with skin and joint epitopes (41). The generation of autoimmune responses is directly correlated with immune susceptibility related to leukocyte antigens (42). In UC, prolonged course of the disease, reduced quality of life, intestinal fibrosis, and increased incidence and mortality are strongly associated with untreated EIMs. The gut is the preferred site for the treatment of EIMs. Active treatment of EIMs in early-stage UC has now been shown to be effective in preventing catastrophic outcomes.

## UC model construction methods and evaluation criteria

### Chemically induced models

#### DSS-induced UC model

DSS is a polyanionic derivative that appears as a white powder, is odorless, and gives a clear solution when dissolved in water. It is one of the most common and effective drugs for UC induction (2). In DSS-induced intestinal inflammation generally occurs in the lamina propria of the intestine (43). Sulfated polysaccharides in DSS exert a toxic effect on the colonic epithelium, which disrupts the epithelial cell barrier of the intestine, leads to epithelial injury and loss of mucosal barrier integrity and induces immune responses (44). DSS can induce UC by affecting host DNA replication, inhibiting the overproliferation of intestinal epithelial cells, enhancing the release of inflammatory factors, and disrupting the balance of the gut microbiome (45). The partial pathogenic factors are shown in Table 1.

**Table 1:** Pathogenic factors of DSS-induced UC.

Test animal	Pathogenic conditions	Influencing factors
Mouse	Susceptibility gene	CARD15 (59)
	Cytokine	TNF-*α*, IL-1β (60, 61), INF-*γ* (62)
	Toll-like receptor	NOD2 (63), Flagellin/TLR5 (64)
	Adhesion molecule	ICAM1 (65), CD98 (66), VCAM-1 (67)
	T cell	TH17 cell (68)

Generally speaking, high molecular weight DSS does not lead to colitis, whereas low molecular weight (5 KDa) DSS induces inflammation (43). DSS is not easily soluble in water, so DSS is added to distilled water heated to 37°C in advance. The DSS aqueous solution should be stirred thoroughly with a sterilized glass rod to ensure that DSS is completely dissolved before administration. If DSS is not sufficiently dissolved, the undissolved salt will block the mouth of the bottle, resulting in difficulty in drug administration or insufficient drug intake of the animals, and thus failure of the model construction. It is also necessary to check the clarity of the liquid before administration. Cloudiness may indicate microbial contamination; Fresh DSS solution should be prepared under sterile conditions. In this case, the bottle should be cleaned or replaced with a new one to prepare new DSS aqueous solution. This is very important. Careless check may lead to wrong test results. While the test group is administered the drug, the control group should be given the same quality of water.

During the test period, some points are worth paying special attention to, as follows.

- On Day 0, the mice in the administration group and the control group should be marked with a marking pen. This is to avoid potential inaccuracy of the subsequent test results, as earmarking may cause stress in the animals, affecting their normal feeding and drinking.
- To avoid fighting between the test mice (which may cause their injuries and thus affect the test results), the mice with gentle disposition should be selected and reared in separate cages to participate in the test.
- The administration method needs to be determined. Rectal administration may lead to stress reaction in mice, intragastric administration may lead to death of mice due to incorrect operation, and free drinking may lead to insufficient drug intake. Therefore, the determination of the administration method is an important factor influencing the test results.
- During the first 3 days of the test, the weight of the mice in the administration group may increase, and start to gradually decrease with the occurrence of bloody stools. Since there is no clear requirement on when to sacrifice the mice, the investigators can determine when to sacrifice the mice for the subsequent test according to the weight loss or the severity of bloody stools (46).

#### TNBS-induced UC model

2,4,6-Trinitrobenzenesulfonic acid (TNBS) is a hapten that becomes immunogenic after binding to host proteins. TNBS induces an immune response in the host when it binds to the host. The model induced with TNBS is characterized by short modeling time, reproducibility, and simplicity of induction. TNBS is a typical chemical substance used to induce ulcerative colitis models. The mechanism of TNBS as an inducer is as follows: TNBS acts as a hapten, penetrating ethanol-damaged mucosa and binding to host proteins to form neoantigens that trigger a Th1-dominant immune response (47). This model is an immune response mediated primarily by the helper T cell TH1, characterized by infiltration of CD4 + cells, neutrophils, and macrophages, which ultimately leads to transmural colitis (43).

When TNBS is used as an inducer, ethanol is commonly used as its solvent, and the administration is generally intrarectal. Ethanol can first disrupt the intestinal mucosal barrier and assist TNBS in causing intestinal inflammatory injury (48, 49). However, there is no consensus among scholars on the choice of TNBS and ethanol doses. Park et al. (50) used 0.8 mL of 5% TNBS as an inducer and 50% ethanol solution as a solvent, while Isik et al. (51) used 1 mL of 3% TNBS as an inducer and 40% ethanol as a solvent.

### Induction of UC by gene knockout technology

The use of genetic engineering technologies for model establishment is an important turning point in the research of UC (52). In the 1990s, Kühn R and his team were the first to propose the construction of a colitis model by knocking out the gene that interferes with the production of IL-10 in the mouse. The emergence of this technology was reported as the establishment of the first spontaneous colitis mouse model (53). Polymorphisms in the IL-10 gene are associated with increased UC susceptibility (54), and genetic polymorphism at the IL-10 gene locus promotes the development of ulcerative colitis (55). Anderson C et al. demonstrated that IL-10 gene knockout mice would develop spontaneous colitis 3 months after knockout (56), with inflammation concentrated in the duodenum, proximal jejunum, and ascending colon (53). The IL-10 gene knockout-induced colitis model is mainly characterized by massive infiltration of macrophages, neutrophils, and lymphocytes (53). Some scholars found that the mice subject to knockout of MUC2 and IL-10 genes showed shortened time to disease and more severe symptoms compared with the mice receiving knockout of IL10 gene. Such a model constructed by double-knockout technology is suitable for the study of UC. Dual knockout mice lacking MUC2 and IL-10 developed severe colitis, growth retardation, bloody diarrhea, and intense mucosal inflammation at 4 weeks of age – very similar to human UC. Thus, the model established by this technology was able to mimic the clinical symptoms of UC patients (57). In addition, deletion of the IL-10 gene in T-cells, especially in regulatory T-cells, contributes to the development of colitis. Therefore, IL-10 plays a critical role in intestinal homeostasis (58). Other UC-relevant models include oxazolone-induced colitis, which models Th2-dominated inflammation, and adoptive T-cell transfer models, which are valuable for dissecting chronic adaptive immune responses.

### Model evaluation criteria

The UC models constructed through different methods have different evaluation criteria, as shown in Table 2.

**Table 2:** Evaluation indicators for different UC models.

Model type	Evaluation dimensions	Specific indicators
DSS model	Clinical indicators	Weight loss rate, bloody stool score (0–4 points), diarrhea severity score (0–3 points)
	Pathological indicators	Degree of crypt destruction and grading of inflammatory cell infiltration (mucosal/submucosal)
Gene Knockout model	Molecular biology indicators	The mRNA expression levels of IL-10 and TNF - α, as well as the degree of neutrophil infiltration detected by immunohistochemistry

## Limitations and future directions

At present, despite their utility, current UC animal models have important limitations due to differences in induction mechanisms, species biology, and lack of standardization. For example, chemically induced models still rely on exogenous chemical substances to damage animal intestinal mucosa, which is different from the natural onset of UC. For instance, DSS-induced models rely on chemical injury to the epithelium and produce short-term acute inflammation, whereas UC in humans is typically chronic and relapsing. Acute inflammation is its main pathological feature, while the pathological process of UC is usually chronic and recurrent; TNBS requires ethanol pre-treatment to facilitate haptenation, resulting in a Th1-dominated response more typical of Crohn’s disease than UC, which is confined to the mucosa and associated with Th2/Th17 profiles. In addition, the degree of inflammation development in chemically induced models is greatly affected by external factors such as drug concentration, animal species, and gender. Different methodologies also lead to significant differences in the results, and the standardization level is relatively insufficient.

Therefore, based on the limitations of existing UC models, in the future, we need to consider multiple factors and precision when constructing UC pathological models. A more comprehensive modeling approach could combine chemical induction with gene editing and microbiota manipulation to better recapitulate the pathogenic cascade---from epithelial barrier disruption to immune dysregulation and microbial imbalance.

At present, the UC model has laid a certain foundation for exploring the pathogenesis and related drug development, but it still has certain limitations. Further integration of biotechnology—including gene editing, organoid systems, and microbiome transplantation—may improve model fidelity and enable more accurate simulation of UC pathogenesis. This, in turn, could accelerate translation from model systems to clinical therapies.

## Conclusion

Against the background of the development of new drugs and the prevention and intervention of such diseases, the development and refinement of UC models remains central to preclinical research and drug discovery. Despite their widespread use in human medicine, these models remain underutilized in veterinary research. Improved model systems can advance translational applications across species.
